# Supplementary figures and images for: Specific facial signals associate with categories of social actions conveyed through questions
Source: PLoS One. 2023 Jul 19;18(7):e0288104. doi: 10.1371/journal.pone.0288104 (PMC10355412; doi:10.1371/journal.pone.0288104)

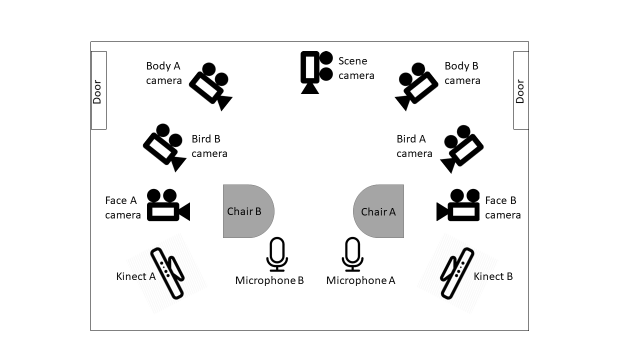

Supplement: S1 Appendix — [33]. (TIFF) [file pone.0288104.s001.TIFF]
