# Supplementary material for: Specific facial signals associate with categories of social actions conveyed through questions
Source: PLoS One. 2023 Jul 19;18(7):e0288104. doi: 10.1371/journal.pone.0288104 (PMC10355412; doi:10.1371/journal.pone.0288104)
Supplement: S2 Appendix — (DOCX) [file pone.0288104.s002.docx]

**S2 Appendix B. Summary of the relationship between the 34 CoAct dyads and their conversation quality.**

| **Min length relationship** | **Mean closeness**^a^ | **Mean conversation quality** | **Platonic relationship**^b^ |
| --- | --- | --- | --- |
| 4 months | 5.98 | 6.15 | “yes” = 57  “no” = 11 |

*Note.* The scale for closeness and conversation quality ranged from 1 (not close/poor conversation quality) – 7 (very close/excellent conversation quality).

^a^ Because one speaker from a dyad did not completely fill in the questionnaire, there is one missing value for Mean closeness.

^b^ One dyad differed in their response to the question whether they shared more than a platonic relationship.
