# Supplementary material for: Specific facial signals associate with categories of social actions conveyed through questions
Source: PLoS One. 2023 Jul 19;18(7):e0288104. doi: 10.1371/journal.pone.0288104 (PMC10355412; doi:10.1371/journal.pone.0288104)
Supplement: Attachment — Submitted filename: Review.docx [file pone.0288104.s003.docx]

**Reviewer’s Comment**

This research investigates the association between different facial signals and social actions expressed through questions. The research questions that were asked were:

1) **RQ1:** Do different social actions result in a different distribution of facial signals?
2) **RQ2:** How does the timing of facial actions coordinate with verbal utterances in performing social actions?
3) **RQ3:** In what way do the facial signals of different social actions differ in terms of their temporal organization?

Specific hypotheses that were asked were:

**H1:** Social actions will differ in accordance with the facial signals that accompany them.

**H2:** Facial signals are most likely to occur at the beginning of a conversation.

Based on the results presented, the following were the observations presented for different RQs:

1) **RQ1 response:** The facial signals distribute differently across social signals.
2) **RQ2 response:** The onset of the facial signal was most common at the moment when the verbal utterance began.
3) **RQ3 response:** The temporal organization of the facial signal sequences was distributed differently across social actions.

—------------------------------------------------------------------------------------------------------------------

Overall, the paper is well-written and presents a clear and concise argument related to different facial signals associated with different social actions. The methodology is appropriate, the results are statistically significant. The paper makes an important contribution to the existing body of knowledge on the topic. I can also see a wider applicability of the research when having one-on-one conversations and preparing oneself beforehand to come up with appropriate responses.

There are still some comments that need to be addressed for the paper to be published. Here are my comments:

1) In light of the fact that this experiment involved the capture of the faces of participants using cameras and informed consent was obtained from the participants informing them that their facial signals or facial profiles would be captured, how did the author account for the Hawthorne effect? Did the authors consider the impact of the experiment on the accuracy of the data collected?
2) Presently, the study has a skewed distribution of gender, i.e., 51 females and only 17 males. Additionally, the standard deviation is high, i.e., 08 years.
a) Has this high SD affected the timing and distribution of facial signals across social actions?
b) How did the authors account for the gender-based differences in facial signals between males and females, given that literature has found gender differences in emotional expression through the face [1,2]? It is understandable that the component measured here was facial signals and not facial expressions; however, facial signals also include muscle movements that may differ between genders. I believe facial signals will vary across genders.

[1] <http://ccat.sas.upenn.edu/plc/communication/soojin.htm>

[2] <https://www.frontiersin.org/articles/10.3389/fpsyg.2015.01372/full>

3) Furthermore, why did the authors not examine dyadic conversions between?
   a) Male-male diads
   b) Male-female diads
   c) Female-female diads

This question is intended to assess the generalizability of the study to a broader population. It would have been interesting to see the timing and distribution of the facial signals across social categories between these genders.

4) In your lines 218 to 220 - “Following the same procedure as for questions transcriptions, interrater reliability for the social action categories was calculated for 10% of the total number of question annotations (n = 686)”.

Considering that the total number of annotated questions was 2082 (lines 205-206), 10% of that number should represent something else? I believe that this needs to be corrected. Or correct me if I am wrong.

5) In line (353) you mentioned that you used holdout cross-validation. How were your training and validation datasets distributed? You should explicitly mention the ratio of the training and validation dataset somewhere in your paper.
6) Please include verbatims of the verbal utterances for different social actions in sections 4.1, 4.2, and 4.3. By doing so, the audience will have a better understanding of the paper. As an example, what verbatim responses occurred during *other-initiated repairs* during which eyebrows were raised and frowned?
7) Please indicate explicitly in the discussion section how your study validated the two hypotheses you discussed in section 1.1. In doing so, you will make your audience feel connected to the introduction section.

—------------------------------------------------------------------------------------------------------------------
