# Supplementary material for: Specific facial signals associate with categories of social actions conveyed through questions
Source: PLoS One. 2023 Jul 19;18(7):e0288104. doi: 10.1371/journal.pone.0288104 (PMC10355412; doi:10.1371/journal.pone.0288104)
Supplement: Attachment — Submitted filename: Review 2.docx [file pone.0288104.s005.docx]

**Reviewer’s Comment (2)**

**Previous Comment from the Reviewer (1):**

There are still some comments that need to be addressed for the paper to be published.

Here are my comments:

1) In light of the fact that this experiment involved the capture of the faces of participants using cameras and informed consent was obtained from the participants informing them that their facial signals or facial profiles would be captured, how did the author account for the Hawthorne effect? Did the authors consider the impact of the experiment on the accuracy of the data collected?

**Response by the Authors (1)**
We thank the reviewer for raising this point. We believe our results are as close as possible to the natural conversation when recording it (in any setting) since we asked participants to interact freely, resulting in entirely unscripted and unprompted conversation. The participant dyads often ***discussed topics that were very personal or even private, leading us to believe that they felt comfortable enough to behave naturally.***

To address this point, we have added a sentence to our methods section: *“We are confident that the data collected in this corpus reflects common everyday dialogue as participants varied in the content they conveyed and expressed diverse perspectives on comparable themes while being recorded, indicating a high level of comfort.”* (p.8-9, lines 174-177).

Importantly, the information participants received prior to being recording did not mention facial behaviour or any other form of visual behaviour. Instead, the emphasis was on aiming to capture casual conversation, and asked them to ignore the recording devices the best they can. Of course, we cannot rule out that the presence of the cameras may have influenced participants’ visual behaviour to some extent, but since the behaviour looked natural, reproduced certain findings that have been reported in the literature before (e.g. on the correlations between eyebrow movements and questions, or specific combinations of facial signals), we are confident that any such influences would not have led to significant distortions of the data. Moreover, there is no alternative, since due to GDPR guidelines it is not allowed to record participants without their prior consent.

Additionally, we addressed the Hawthorne effect more explicitly in the discussion: “Participants varied significantly in the content they conveyed, as well as their perspectives, which we interpreted to indicate a high level of comfort with being recorded and therefore to be an accurate reflection of common everyday dialogue. However, different social factors could still have an effect on participant’s signalling behaviour, such as the degree of familiarity between speakers, or the fact that the speakers were sitting in a lab while being recorded, potentially causing them to modify their behaviour due to being observed (similar to the ‘Hawthorne effect’ [79]). Corpus data inherently involves many intertwined layers of behaviour, which we cannot tease apart without experimental manipulation. Future experimental studies should therefore investigate the exact contribution facial signals make towards quick social action recognition in conversation, to control for other potential factors (e.g., social factors, turn boundaries, interaction with prosody). Furthermore, investigating visual signalling in other group contexts and across non-WEIRD (Western Educated Industrialized Rich and Democratic) societies would be particularly relevant to find out whether the current findings hold in different social and cultural settings [80,81].” (p. 33-34, lines 689-701).

**Response by the reviewer (1)**

Dear Authors,

I appreciate your thorough response regarding the Hawthorne effect. The measures you took to ensure naturalness in participant behavior are commendable the inclusion of this topic in your discussion was a positive step, and the future research considerations proposed to highlight a much-needed holistic perspective on this issue. Nevertheless, your study prompts us to consider innovative methodologies that could further minimize such observer effects in future research.

One suggestion could be the use of immersive, naturalistic settings that mimic participants' everyday environments, thus providing a less obtrusive context for data collection. Another possibility could be the extension of recording durations, allowing participants to grow accustomed to the recording process and thus possibly mitigating observer effects over time.

In conclusion, while the authors' approach was well-conceived and carefully executed, the complexities tied to the Hawthorne effect remain a thought-provoking challenge for researchers in this field, necessitating creative methodological advancements for its effective mitigation.

—----------------—--------------------------------------------------------------------------------------------

**Previous Comment from the Reviewer (2):**

Presently, the study has a skewed distribution of gender, i.e., 51 females and only 17 males. Additionally, the standard deviation is high, i.e., 08 years.

- Has this high SD affected the timing and distribution of facial signals across social actions?
- How did the authors account for the gender-based differences in facial signals between males and females, given that literature has found gender differences in emotional expression through the face [2,3]? It is understandable that the component measured here was facial signals and not facial expressions; however, facial signals also include muscle movements that may differ between genders. I believe facial signals will vary across genders.

[2] <http://ccat.sas.upenn.edu/plc/communication/soojin.htm>

[3] <https://www.frontiersin.org/articles/10.3389/fpsyg.2015.01372/full>

**Response by the Authors (2)**

We agree that people vary a lot in visual signal production and perception, which may indeed translate to differences in conversational facial signalling. However, we feel that emphasizing gender differences would potentially reinforce gender stereotypes [1], which we would very much prefer to avoid in light of the current gender debates, which conceives of gender as a dimension (or in fact several) rather than a categorical construct. Additionally, at the time that the corpus data was being collected, gender was still defined as a binary construct, which may not accurately reflect the way all of the participants identify. Therefore, we do not feel that our data would allow a meaningful in-depth analysis into gender differences.

To address the fact that more intensive research is needed to drill deeper into the patterns we have uncovered here by looking at different social factors (such as familiarity or similarities between speakers or other individual differences across speakers), we have added a sentence to the discussion, for which we refer the reviewer to our response to point 1. However, an in-depth analysis that does justice to these different social factors would go beyond the current study.

[1] <https://doi.org/10.1146/annurev-psych-122216-011719>

**Response by the reviewer (2)**

Dear Authors,

Your response acknowledging the multifaceted nature of gender in today's society is deeply appreciated. The nuanced understanding exhibited in your study adds a layer of depth that demonstrates your comprehensive grasp on the subject matter.

While I understand your desire to avoid reinforcing gender stereotypes, it may be beneficial to your audience to lightly touch on the gender imbalance in your participant group. The inclusion of such a discussion could offer a more holistic view of the potential variables at play in your study.

Your decision to delve into the impact of social factors, such as speaker familiarity and individual differences, adds an intriguing dimension to your research. However, it does bring up a point of methodological interest. If participants were selected due to certain shared traits or relationships, this would align with 'purposive sampling' methods. If that is the case, it might be useful to clarify this in your methodology section for the benefit of your readers.

—---------------------------------------------------------------------------------------------------------------

**Previous Comment from the Reviewer (3):**

In your lines 218 to 220 - “Following the same procedure as for questions transcriptions, interrater reliability for the social action categories was calculated for 10% of the total number of question annotations (n = 686)”. Considering that the total number of annotated questions was 2082 (lines 205-206), 10% of that number should represent something else? I believe that this needs to be corrected. Or correct me if I am wrong.

**Response by the Authors (3)**

We have adjusted the sentence to correctly represent the number of annotations and to clarify the methodology (p. 11, lines 225-230). “A subset of 2082 questions were coded for their social action category. The detailed coding scheme for the social action categories was developed for a larger project that the present study is part of, and was inspired by a combination of previous categorizations [10,12,58,65,66]. We took into account the sequential position and form of the social actions in conversation, state of the common ground between speakers, communicative intention, as well as the result of the speaker’s utterance on the addressee. This resulted in eight discrete social action categories”

**Response by the reviewer (3)**

Thank you.

—-------------------------------------------------------------------------------------------------------------

**Previous Comment from the Reviewer (4):**

In line (353) you mentioned that you used holdout cross-validation. How were your training and validation datasets distributed? You should explicitly mention the ratio of the training and validation dataset somewhere in your paper.

**Response by the Authors (4)**

We used the default value for distributing the training and validation datasets. Therefore, the fraction of data to keep for training was 0.8. We have added the following sentence: “We used the default value for distributing the training and validation datasets, resulting in a data fraction of 0.8 for the training dataset, and 0.2 for the validation dataset.” (p. 20, lines 392-394).

**Response by the reviewer (4)**

Thank you.

—-------------------------------------------------------------------------------------------------------------

**Previous Comment from the Reviewer (5):**

Please include verbatims of the verbal utterances for different social actions in sections 4.1, 4.2, and 4.3. By doing so, the audience will have a better understanding of the paper. As an example, what verbatim responses occurred during other-initiated repairs during which eyebrows were raised and frowned?

**Response by the Authors (5)**

We have repeated the examples of the social action categories in the discussion to make interpretation of the results more comprehensible.

**Response by the reviewer (5)**

Ok

—---------------------------------------------------------------------------------------------------------------

**Previous Comment from the Reviewer (6):**

Please indicate explicitly in the discussion section how your study validated the two hypotheses you discussed in section 1.1. In doing so, you will make your audience feel connected to the introduction section.

**Response by the Authors (6)**

We have added a more explicit description of these validations by adding the following sentences: “Our results presented above therefore validate our first hypothesis that social actions would differ with respect to the facial signals they are associated with.” (p. 30, lines 620-621),

**Response by the reviewer (6)**

Thank You
